# Psychiatric adverse events and effects on mood with prolonged-release naltrexone/bupropion combination therapy: a pooled analysis

**DOI:** 10.1038/s41366-018-0302-z

**Published:** 2019-01-21

**Authors:** Xavier Pi-Sunyer, Caroline M. Apovian, Susan L. McElroy, Eduardo Dunayevich, Lisette M. Acevedo, Frank L. Greenway

**Affiliations:** 1 Columbia University Medical Center, New York, NY, USA; 2 Boston University School of Medicine and Department of Medicine Section of Endocrinology, Diabetes and Nutrition, Boston Medical Center, Boston, MA, USA; 3 Lindner Center of HOPE, Mason, and Department of Psychiatry and Behavioral Neuroscience, University of Cincinnati College of Medicine, Cincinnati, OH, USA; 4 Annexon Biosciences, South San Francisco, CA, USA; 5 Nalpropion Pharmaceuticals, Inc, La Jolla, CA, USA; 6 Pennington Biomedical Research Center, Louisiana State University, Baton Rouge, LA, USA

## Abstract

**Background/objectives:**

Prolonged-release (PR) naltrexone 32 mg/bupropion 360 mg (NB) is approved for chronic weight management as an adjunct to reduced-calorie diet and increased physical activity. Central nervous system-active medications have the potential to affect mood; therefore, post hoc analysis of clinical trial data was conducted to evaluate psychiatric adverse events (PAEs) and effects on mood of NB therapy versus placebo.

**Subjects/methods:**

Data were pooled from 5 prospective, double-blind, randomized, placebo-controlled clinical trials (duration range, 24–56 weeks) of NB in subjects with overweight or obesity. PAEs were collected via AE preferred terms, organized into major subtopics (e.g., anxiety, depression, sleep disorders), and divided into category terms (e.g., anxiety, potential anxiety symptoms). Additionally, the Inventory of Depressive Symptomatology Self Report (IDS-SR; score range 0–84) and the Columbia Classification Algorithm of Suicide Assessment (C-CASA) evaluated treatment-emergent depressive/anxiety symptoms and suicidal behavior/ideation, respectively.

**Results:**

Baseline characteristics and comorbidities were comparable for placebo (*n* = 1515) and NB (*n* = 2545). Most common PAEs in the NB group (using category grouping; NB vs placebo) were sleep disorders (12.7 vs 7.9%, *P* < 0.001), anxiety (5.4 vs 3.3%, *P* = 0.029), and depression (1.8 vs 2.7%, *P* = 0.014); *P*AEs were more frequent during dose escalation and generally mild or moderate. Mean (SD) changes in IDS-SR total score from baseline to endpoint were small in both groups: 0.13 (5.83) for NB and −0.45 (5.65) for placebo. Retrospective AE categorization via C-CASA confirmed no completed suicides, suicide attempts, or preparatory acts toward imminent suicidal behavior.

**Conclusions:**

This large pooled analysis of 5 clinical trials provides additional safety information about the NB PAE profile. Anxiety and sleep disorder-related PAEs were more frequent with NB versus placebo but were mostly mild to moderate and generally occurred early. Depression-related PAEs were less common with NB than placebo, and NB was not associated with suicidal ideation or behavior in this patient population.

## Introduction

Excess body weight is a risk factor for many serious illnesses, and minor weight loss may reduce risk. For example, each 1-kg reduction in body weight is associated with a 16–33% lower risk of diabetes in overweight, non-diabetic adults [1–3]. Among individuals with obesity (BMI > 30 kg/m^2^) or with type 2 diabetes and overweight (body mass index [BMI] ≥ 25 kg/m^2^), weight loss of 5–10% is associated with clinically relevant improvements in many car-diometabolic risk factors as well as improvement in measures of quality of life, mobility, depression, sexual dysfunction, and urinary stress incontinence [2, 4].

The combination of prolonged-release (PR) naltrexone 32 mg plus PR bupropion 360 mg (NB) is approved worldwide for chronic weight management as an adjunct to a reduced-calorie diet and increased physical activity in adults with an initial BMI of ≥30 kg/m^2^ or ≥27 kg/m^2^ and ≥1 weight-related comorbidity (e.g., dyslipidemia, controlled hypertension, type 2 diabetes mellitus) [5, 6]. Evidence suggests that NB acts on 2 areas of the central nervous system: the mesolimbic reward pathway and the hypothalamic hunger system [7, 8]. Combined data from phase 3 clinical trials showed that NB therapy was associated with significantly greater weight loss compared with diet and exercise alone (7.0 vs 2.3%; *P* < 0.001) [9]. A weight loss of ≥5% over a 56-week study period was achieved by 53% of individuals treated with NB compared with 21% of individuals who received placebo (*P* < 0.001) [9]. The most common adverse events (AEs) associated with NB in clinical trials were nausea, constipation, and headache, which were generally transient [9–13].

Medications that act on the central nervous system have the potential for negative effects on mood [14] and should therefore be closely examined for psychiatric AEs (PAEs). Safety concerns, including increased risk of PAEs such as depression, have hindered the development and approval of centrally acting obesity medications [14, 15]. Postmarketing surveillance of rimonabant demonstrated an increased risk of depressive disorders, leading the European Medicines Agency to withdraw the product and the US Food and Drug Administration to deny approval [14]. In the case of ecopipam, higher rates of PAEs (including depression and suicidal ideation) were observed with ecopipam treatment compared with placebo, leading to discontinuation of the phase 3 clinical studies [15].

The individual components of NB have been approved for use in various indications for more than 30 years (naltrexone to treat opioid and alcohol dependence and bupropion to treat depression, prevent seasonal affective disorder, and aid in smoking cessation) [16–18]. However, given concerns regarding PAEs with other anti-obesity treatments [14, 15], it is important to evaluate the potential for PAEs with NB. Adding further relevance to the study of PAEs with NB, it is also important to recognize that some data have suggested that obesity itself may be associated with depression and suicidal ideation [15, 19]. A meta-analysis of 15 longitudinal studies reported bidirectional associations between depression and obesity over time, finding that individuals who were obese had a 55% increased risk of developing depression and individuals who were depressed had a 58% increased risk of becoming obese [19]. Also, the use of antidepressants may be associated with increased risk of suicidal behavior, particularly in adolescents and young adults; however, a link between antidepressant use and completed suicide has not been conclusively demonstrated and a meta-analysis of 51 trials reported no significant differences in expressed suicidal ideation or behavior in adults with major depressive disorder (MDD) receiving bupropion compared with placebo [20, 21]. In the current report, we evaluate the PAEs, as well as results from a patient-reported outcome measure focusing on depression and anxiety symptoms, in subjects receiving NB therapy or placebo in a pooled analysis across multiple placebo-controlled studies from the NB clinical research development program.

## Subjects and methods

### Study designs

This is a post hoc analysis of data pooled from 5 placebo-controlled clinical trials, including 1 phase 2 trial [22] and 4 phase 3 trials [10–13] of NB in subjects with overweight or obesity. The analysis was limited to subjects randomized to daily doses totaling 32 mg naltrexone PR plus 360 mg bupropion PR (or 32 mg naltrexone immediate release plus 400 mg bupropion PR in study NB-201) [22] or placebo from the following studies: NB-201 (NCT00364871 [ClinicalTrials.gov], excluding the open-label extension period; phase 2) [22], COR-I (NCT00532779, excluding the 2-week discontinuation period; phase 3) [11], COR-BMOD (NCT00456521; phase 3) [13], COR-II (NCT00567255, the NB group included subjects randomized to NB who did not achieve or maintain a ≥5% weight reduction from baseline by week 28 and were switched to daily 48 mg naltrexone PR plus 360 mg bupropion PR in a randomized double-blind manner; phase 3) [10], and COR-Diabetes (NCT00474630, phase 3) [12]. All 5 studies were prospective, double-blind, and randomized and included a dose escalation period of 3 to 4 weeks. Study durations were either 24 weeks (study NB-201) or 56 weeks (phase 3 studies).

### Common criteria for participation across studies

Criteria for participation in the individual studies have been published in detail [10–13, 22]. Key general inclusion criteria across the 4 phase 3 studies were that subjects had to be free of opioid medication for 7 days before randomization and have no clinically significant abnormal laboratory results. In the phase 2 study, subjects were required to have a BMI of 30–40 kg/m^2^. For 3 of the phase 3 studies (COR-I, COR-BMOD, and COR-II), subjects were required to have a BMI of 30–45 kg/m^2^ (subjects with obesity) or a BMI of 27–45 kg/m^2^ (subjects with obesity and controlled hypertension and/or dyslipidemia). In COR-Diabetes, patients were required to have a BMI of 27–45 kg/m^2^ and type 2 diabetes mellitus.

To minimize the risk of mood changes, psychiatric inclusion criteria common across the 4 phase 3 studies were baseline Inventory of Depressive Symptomatology Self-Report (IDS-SR) scores < 2 on items 5 (sadness), 6 (irritability), 7 (anxiety/tension), and 18 (suicidality) and an IDS-SR total score < 30 at screening. The phase 2 study stipulated a score < 11 on the depression and anxiety components of the Hospital Anxiety and Depression scale. Patients were excluded from the 4 phase 3 trials if they had serious psychiatric illness, including lifetime history of bipolar disorder, schizophrenia or other psychotic disorders, bulimia nervosa, or anorexia nervosa; current serious personality disorder, current severe MDD, recent (previous 6 months) suicide attempt or current active suicidal ideation, or recent hospitalization due to psychiatric illness. In addition, subjects were excluded if they were in need of medications for the treatment of a psychiatric disorder (with the exception of short-term insomnia) within the previous 6 months or if they had a history of drug or alcohol abuse or dependence within 1 year. Key exclusion criteria for the phase 2 study were similar (serious psychiatric condition, history of drug or alcohol abuse within 5 years).

### Treatments

Following screening, participants included in this pooled analysis received 32 mg/d naltrexone PR plus 360 mg/d bupropion PR (or 32 mg naltrexone immediate release plus 400 mg of bupropion PR [NB-201]; NB) or a matching placebo administered in divided doses, twice daily [10–13, 22]. Medication was initiated at a daily dose of 8 mg/d naltrexone and 90 mg/d bupropion (100 mg/d in NB-201) that was escalated over 3–4 weeks until reaching the maintenance dose. Study participants received instructions to reduce caloric intake and increase physical activity in all the studies; however, the intensity of the intervention varied across studies.

### Assessment of psychiatric adverse events

For this analysis, PAEs were collected at study visits and coded into AE preferred terms using the Medical Dictionary for Regulatory Activities, version 12.0. PAEs were organized into major subtopics to represent the specific medical concepts of anxiety, depression, sleep disorders, hostility, mood disorders, psychosis, and nonspecific mental disorders. Only subtopics that had an incidence rate of ≥5% in either the NB or placebo group were selected for further evaluation. PAE subtopics of anxiety, depression, and sleep disorders had incidence rates ≥ 5% and were then further divided into category terms (e.g., anxiety, potential anxiety symptoms) for evaluation (Table 1). Treatment-emergent depressive and anxiety symptoms were also assessed in the 4 phase 3 studies via change in the IDS-SR total score and scores for items 5 (sadness), 6 (irritability), 7 (anxiety/tension), and 18 (suicidality). IDS-SR is a 30-item, subject-rated inventory of depressive symptoms, with a 7-day recall period. It is scored from 0 to 84, with a score of 0–13 indicative of no depression; 14–25, mild depression; 26–38, moderate depression; 39–48, severe depression; and ≥49, very severe depression [23]. IDS-SR was assessed at baseline and every 4 weeks through the end of the study. In the Phase 3 trials, subjects who reported treatment-emergent IDS-SR scores ≥ 2 on item 5 [sadness], item 6 [irritability], item 7 [anxiety/tension] or suicidality items, or a total score ≥ 25 (or ≥30 for subjects with a score ≥ 25 at screening) were further evaluated and, if indicated, referred to a psychologist or psychiatrist, as values that met these thresholds may have indicated the presence of a treatment emergent depressive or anxiety disorder. To assess AEs that could represent suicidal ideation or behavior, a retrospective assessment tool, the Columbia Classification Algorithm of Suicide Assessment (C-CASA), was used [24]. Possibly suicide-related AEs were based on the following C-CASA categorizations: no event (code 0), completed suicide (code 1), suicide attempt (code 2), preparatory act toward imminent suicidal behavior (code 3), suicidal ideation (code 4), self-injurious behavior, intent unknown (code 5), not enough information (fatal; code 6), other (no evidence of suicidality or deliberate self-harm; code 8), and not enough information (nonfatal; code 9). To increase the sample size and not miss any important but rare events, the C-CASA was performed on data from subjects receiving all doses of combination naltrexone/bupropion.

### Statistical analysis

Parameters were analyzed in the safety population, which included all randomized subjects who were administered ≥ 1 tablet of study treatment and had ≥1 investigator contact/assessment at any time after the start of study treatment. Analysis of covariance was used to analyze change from baseline in IDS-SR scores, with treatment group and study center as main effects and the baseline measurements as covariates.

All AEs presented are treatment emergent, defined as events that occurred or worsened on or after the date of first dose until 7 days after the last confirmed dose (excluding AEs that occurred during drug discontinuation or extension phases). Overall PAEs and key PAE groupings are presented descriptively. Statistical comparisons of PAEs were made using Cochran-Mantel-Haenszel general association test controlling for study.

## Results

### Study population

#### Baseline demographics and clinical characteristics

Demographic characteristics were similar between the placebo (*n* = 1515) and NB (*n* = 2545) groups in the total pooled safety analysis population, including mean age (approximately 45–46 years), sex (>80% female), and mean BMI (36.2 kg/m^2^; Table 2). Baseline comorbidities, history of anxiety and depression, IDS-SR scores, depression rates (based on IDS-SR score), and use of tobacco and alcohol were also comparable between the placebo and NB groups (Table 2). Although an IDS-SR cut-off score of 30 was used as a criterion for entry into the Phase 3 studies, 6 NB-treated (0.02%) and 2 placebo-treated (0.01%) subjects with baseline IDS-SR scores > 30 were enrolled in the phase 3 studies because their scores had been <30 at screening visits.

#### Subject disposition

Across the studies, 55.0 and 54.7% of subjects assigned to NB and placebo, respectively, completed the treatment. The most common reason for study discontinuation was AEs (23.7% NB, 12.0% placebo), followed by withdrawn consent (7.9% NB, 12.7% placebo) and lost to follow-up (6.5% NB, 9.6% placebo). The mean (SD) number of weeks that subjects received double-blind treatment was 36.4 (23.7) for NB (*P* = 0.11) and 37.6 (21.6) for placebo.

### Outcomes

#### Psychiatric-related AEs

Overall, PAEs based on preferred terms occurred more frequently with NB (22.2%) than placebo (15.5%). In the NB versus placebo groups, the most common (i.e., occurring in ≥2% of subjects in the NB group) treatment-emergent AEs, based on preferred terms in the psychiatric disorders system-order class, were insomnia (9.2 vs 5.9%), anxiety (4.2 vs 2.8%), and irritability (2.6 vs 1.8%; Table 3). The PAE of depression (preferred term) was less common in the NB group compared with the placebo group (0.9 vs 1.5%), and suicidal ideation was uncommon in both groups (<0.1 vs 0.2%, respectively).

When preferred term PAEs were grouped into subtopics (Table 1), the percentage of subjects reporting ≥ 1 of these PAEs for the NB versus placebo groups was 6.1 versus 4.4% for the subtopics of anxiety (*P* > 0.05), 6.3 versus 5.9% for depression (*P* > 0.05), and 13.8 versus 8.4% for sleep disorders (*P* < 0.001). Since the incidence rates in either NB or placebo group were ≥5%, these subtopics were divided into categories for further evaluation (Table 1). Only the categories of anxiety, depression, and sleep disorders had significant differences in incidence rates between NB and placebo (Table 3). The percentage of subjects reporting ≥ 1 of these PAEs in the NB versus placebo groups was 5.4 versus 3.3% for anxiety (*P* = 0.029); 1.8 versus 2.7% for depression (*P* = 0.014); and 12.7 versus 7.9% for sleep disorders (*P* < 0.001). In contrast, for the PAE subtopics of hostility, mood disorders, psychosis, and nonspecific mental disorders, incidence rates in both the NB and placebo groups were <4%; therefore, these subtopics were not examined further.

**Fig. 1:**
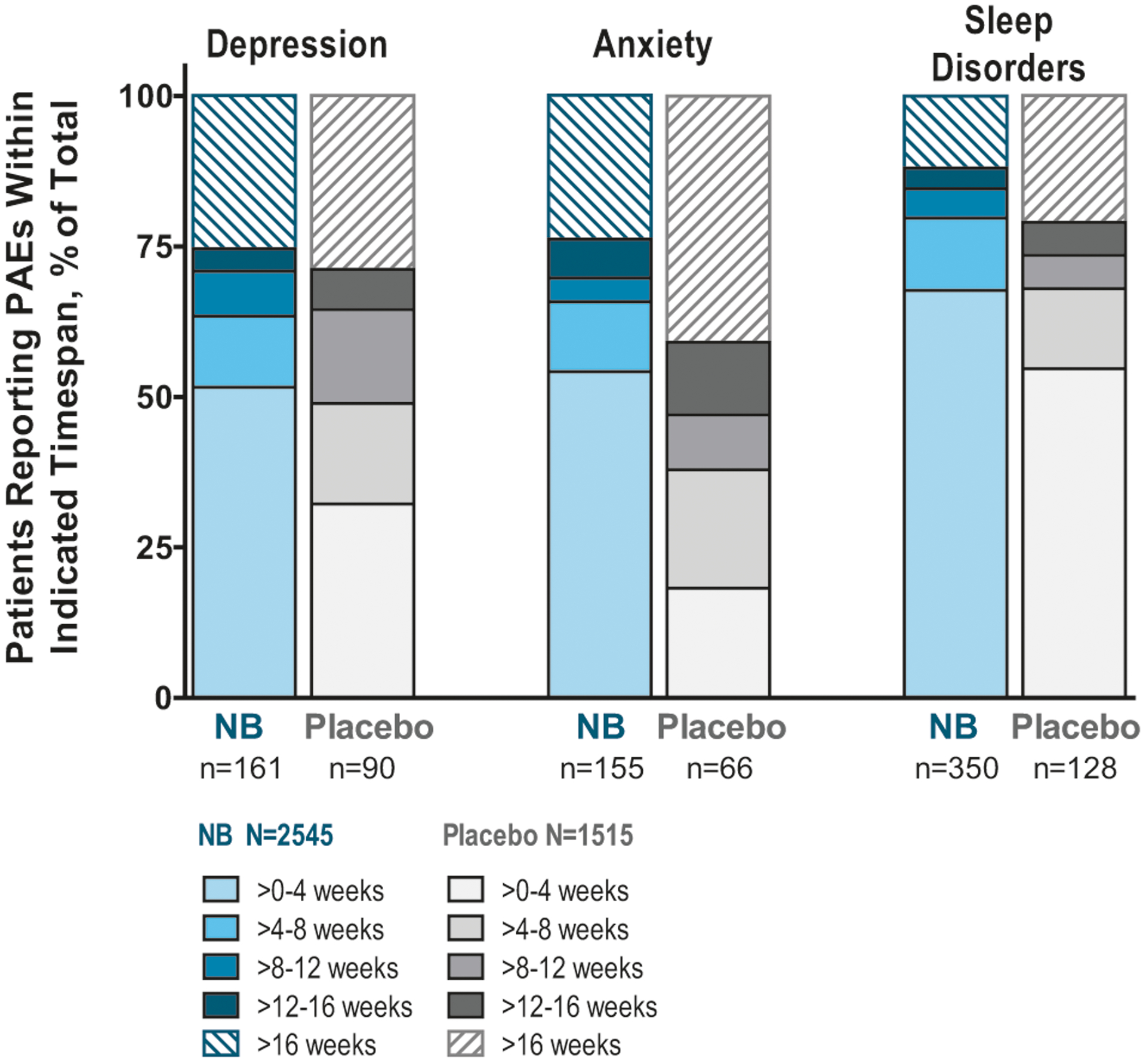
Participants reporting PAEs by treatment week for major subtopics of anxiety, depression, and sleep disorders. Data are presented as percent of participants reporting PAEs for each subtopic. The total number of NB or placebo patients reporting PAEs for each subtopic are listed below each bar. NB, 32 mg naltrexone PR plus 360 mg bupropion PR (or 32 mg naltrexone immediate release plus 400 mg bupropion PR in study NB-201); *PAE* psychiatric adverse event, *PR* prolonged release

PAEs for the subtopics of anxiety, depression, and sleep disorders generally occurred early in the studies, often during the dose escalation period, with the majority being mild or moderate in severity and transient in nature. The percentages of participants reporting onset of symptoms by treatment week for these subtopics are shown in Fig. 1. The median duration of PAEs for NB versus placebo was 4.0 weeks versus 5.0 weeks (depression), 3.0 weeks versus 4.0 weeks (anxiety), and 5.0 weeks versus 6.0 weeks (sleep disorders).

#### Serious PAEs and PAEs leading to study/treatment discontinuation

One serious PAE, anxiety symptoms in a subject receiving NB, occurred during the placebo-controlled studies. PAEs resulted in study discontinuation in 3.0% of placebo subjects and 3.3% of NB subjects (*P* > 0.05). The most frequently reported PAEs (by preferred term, NB vs placebo) that led to study discontinuation were insomnia (0.7 vs 0.5%), anxiety (0.7 vs 0.7%), and depression (0.4 vs 0.9%; all *P* > 0.05).

#### IDS-SR change in total score

**Fig. 2:**
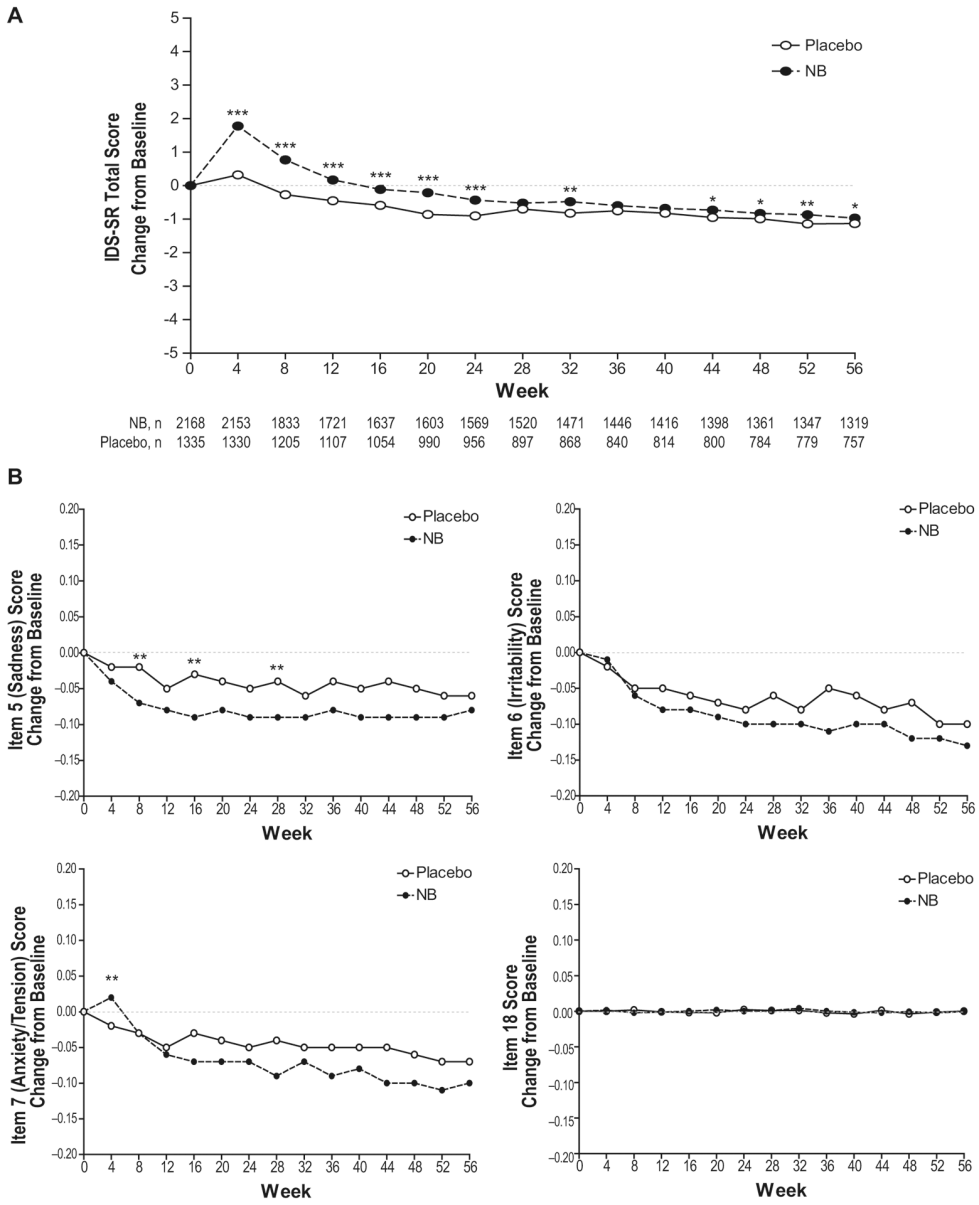
Change in IDS-SR scores^a^ for NB versus placebo, safety analysis set (pooled Phase 3 studies only). **a** Data are mean change from baseline in total IDS-SR score over 56 weeks. **b** Data are mean change from bsaeline for IDS-SR select indvidual items: item 5 (sadness), item 6 (irritability), item 7 (anxiety/tension), and item 18 (suicidality). IDS-SR, Inventory of Depressive Symptomatology, Subject-Rated; NB, 32 mg naltrexone PR plus 360 mg bupropion PR (or 32 mg naltrexone immediate release plus 400 mg bupropion PR in study NB-201); PR, prolonged release. ^a^IDS-SR was not assessed in the phase 2 study (NB-201). *P < 0.05; **P < 0.01; ***P < 0.001 vs placebo

Baseline IDS-SR scores were similar between groups (Table 1). The majority of subjects had IDS-SR scores ≤ 13 indicative of no depression at baseline (88% NB; 89% placebo). The mean (SD) change from baseline at endpoint was 0.13 (5.83) for NB and −0.45 (5.65) for placebo (*P* = 0.004). During the course of treatment, mean IDS-SR total score increased slightly from baseline during the first 12 weeks of NB treatment and subsequently decreased from baseline from week 16 through the end of the studies (Fig. 2). Across the individual studies, review of IDS-SR individual item data indicated that the small treatment effect (i.e., increase) observed in the IDS-SR total score was not due to effects on mood or anxiety items (items 5 [sadness], 6 [irritability], 7 [anxiety/tension], and 18 [suicidality]; Fig. 2), but rather was primarily due to the items measuring appetite, weight, constipation/diarrhea, and other somatic symptoms (data not shown).

#### IDS-SR assessment of treatment-emergent depressive and anxiety symptoms

Treatment-emergent symptoms of depression (i.e., ≥1 postbaseline score of ≥2 for IDS-SR item 5 [sadness] at any time during the study) were reported in 3.5% of NB-treated subjects, whereas symptoms of anxiety (i.e., ≥1 postbaseline score of ≥2 for item 7 [anxiety/tension]) were reported in 6.4% of NB-treated subjects, compared with 3.4 and 4.8%, respectively, of subjects in the placebo group. No differences were observed in proportions of NB- and placebo-treated subjects with ≥1 postbaseline IDS-SR score of ≥25 or with ≥1 postbaseline score of ≥30 among subjects with baseline score ≥ 25 (Table 4).

#### Suicidal ideation

There were no suicides in this pooled analysis, and only 1 death (myocardial infarction) was reported. A retrospective analysis of these pooled data using the C-CASA categorization further demonstrated no completed suicides, suicide attempts, or preparatory acts toward imminent suicidal behavior in any treatment group. Four events of suicidal ideation or behavior were reported: 1 event in the total NB group (n/N = 1/3239, <0.1%) compared with 3 events in the placebo group (n/N = 3/1515, 0.2%). Therefore, the incidence of suicidality (C-CASA codes 1, 2, 3, and 4 combined) was 1 subject (<0.1%) in the total NB group compared with 3 subjects (0.2%) in the placebo group. The overall odds ratio for suicidal ideation or worse for NB compared with placebo was 0.14, suggesting no treatment difference for suicidal behavior. The overall risk difference between treatment groups (NB − placebo) was −0.0018, which further supports the null hypothesis of no treatment effect on incidence of suicidal ideation or behavior in this patient population.

## Discussion

Previous analyses have shown that treatment with NB is associated with significantly greater weight loss compared with diet and exercise alone and that NB is generally well tolerated, with AEs that are generally transient and occur early in treatment [9–13, 22]. The current report provides a detailed analysis of PAEs. Compared with placebo, anxiety-and sleep disorder-related PAEs were more frequent with NB, whereas the frequency of depression-related PAEs was lower in the NB group and NB was not associated with increased suicidal ideation. PAEs were primarily mild to moderate and tended to occur early in the trial during the dose-escalation phase. In addition, the rates of discontinuation due to PAEs were similar between the NB and placebo groups. Consistent with these findings, no meaningful differences in IDS-SR scores were observed between NB and placebo.

These data have potential implications for clinical practice. Given the link between obesity and depression [15, 19], it is reassuring that NB treatment was not associated with a statistically significant increase in depression-related PAEs. In fact, when focusing on the category of depression and excluding broader potential depression-related PAEs (such as irritability and middle insomnia), NB was associated with a significantly lower incidence of depression than placebo treatment. Albeit this population of patients did not have active major severe depression, largely did not have a history of depression, and did not include patients receiving treatment with antidepressants. Consistent with these results, an exploratory open-label study in adults (*N* = 25) with overweight or obesity with MDD demonstrated that NB treatment plus dietary and behavioral counseling was associated with a >50% reduction in depression symptom severity by week 6 that was sustained through week 24; these changes occurred in parallel with clinically meaningful weight loss [25]. Additional information beyond what was observed in these pooled analyses also comes from a large (*N* = 8905) study that assessed the effects of NB on adverse cardiovascular events in subjects with overweight or obesity and cardiovascular risk factors [26]. Subjects with controlled depression and/or who were currently using antidepressant medications were permitted to enroll in this study. The data from this study indicated that an AE of depression resulting in discontinuation was uncommon and comparable between NB and placebo, occurring in only 0.1 and 0.2% of subjects, respectively (*P* = 0.28).

The current analysis provides PAE-focused safety data associated with up to 56 weeks of NB treatment. Additional long-term safety data are available from a randomized open-label study that compared NB plus comprehensive lifestyle intervention with usual care in adults with obesity for 78 weeks [27]. In this study, neuropsychiatric side effects that led to discontinuation of NB overall included anxiety (2.1%) and insomnia (1.2%). These collective data support the finding that depression and suicidality occur infrequently with NB treatment and are also consistent with the slightly increased risks of anxiety and sleep disorders identified in the present report. Taken together, the existing evidence suggests that NB may be associated with increased symptoms of anxiety and sleep disorders, but that these symptoms are likely to be mild or moderate in severity and to occur transiently during the early stages of treatment.

Because NB contains bupropion, and because antidepressant medications have been associated with increased suicidal behaviors and thoughts in some patients [20], the NB prescribing information includes a boxed warning relating to the possibility of emerging suicidal thoughts and behaviors particularly in children, adolescents, and young adults. Other anti-obesity medications targeting the central nervous system (e.g., lorcaserin, liraglutide) also carry warnings pertaining to suicidal thoughts or behaviors or other psychiatric complications [28, 29]. Of note, a post hoc analysis of neuropsychiatric safety data from the liraglutide weight management program reported AEs of suicidal ideation or behavior in 0.3% of patients receiving liraglutide 3.0 mg compared with 0.1% in the placebo group [30]. In this same study, PAEs of depression, anxiety, and insomnia were low (≤3.6%) in both the liraglutide and placebo groups. Prescribing information for phentermine/topiramate ER shows that depression (as well as insomnia and anxiety) PAEs were more common in the highest dose group versus placebo [31]. These warnings underscore the need for studies such as this one investigating risks of PAEs with anti-obesity medications, as well as the need for future studies of PAEs associated with NB in patients with existing depression.

A key limitation of the current report is that the majority of patients did not have psychiatric illness. Indeed, the majority did not have clinically significant depressive symptoms at baseline and could not be using antidepressants. This could be important because of the possibility of drug-drug interactions between antidepressant medications and NB [32], particularly in patients also being treated for depression. Thus, findings from this pooled analysis may not be applicable to patients with mood or other psychiatric disorders. Another limitation, particularly regarding the lack of effect of NB treatment on suicidal ideation, is that relatively few individuals enrolled in these studies were <25 years old, and meta-analyses have revealed that antidepressant medications are less likely to lead to increased suicidal ideations or behavior in patients over 25 years [20]. Nonetheless, in this pooled analysis of a large population of subjects with overweight or obesity who received NB using similar treatment regimens, the risk of PAEs, particularly depression, was low.

## Conclusion

This large, pooled analysis from five clinical trials of NB in subjects with overweight or obesity found that PAEs were primarily mild to moderate and tended to occur during dose escalation. PAEs in the anxiety and sleep disorder categories were more frequent in the NB group compared with the placebo group, whereas the PAE of depression was more frequent with placebo than NB treatment. Notably, NB treatment was not associated with increased suicidal ideation or behavior compared with placebo. These data provide additional safety information and reassurance regarding the psychiatric effects of NB; however, in accordance with the current label, patients should be monitored for depression or suicidal thoughts. More work is needed to evaluate the effect of NB on patients with overweight or obesity and mild to moderate depressive symptomatology, and in patients using antidepressant medications, to elucidate and separate effects of weight loss on depression versus drug effects. This reanalysis serves to provide reassurance that the overall psychiatric safety profile of NB is acceptable.

## Figures and Tables

**Table 1:** PAEs were organized into major subtopics representative of specific medical concepts and categorized according to preferred terms

Major subtopic	Category	PAE preferred terms^a^
Anxiety	Anxiety	Anxiety, nervousness, tension, panic attack, fear, panic reaction, hyperventilation, generalized anxiety disorder
	Potential anxiety symptoms	Stress, restlessness, acute stress disorder, social avoidant behavior, hypervigilance, social phobia
Depression	Depression	Depression, depressed mood, dysthymic disorder, major depression, suicidal ideation
	Potential depression symptoms	Irritability, middle insomnia, libido decreased, stress, poor quality sleep, tearfulness, psychomotor hyperactivity, apathy, crying, depressive symptom, anhedonia, bereavement reaction, emotional distress, loss of libido, negative thoughts
Sleep disorders	Sleep disorders	Insomnia, sleep disorder, abnormal dreams, middle insomnia, poor quality sleep, initial insomnia, nightmare, sleep apnea syndrome, terminal insomnia
	Somnolence	Somnolence, sedation, hypersomnia

*PAE* psychiatric adverse effect

a Coded using the Medical Dictionary for Regulatory Activities, version 12.0

**Table 2:** Baseline demographic and clinical characteristics, safety analysis set

	NB (*n* = 2545)	Placebo (*n* = 1515)
Mean (SD) age, y	45.9(11.2)	45.3 (11.4)
Age 18–44 y, *n* (%)	1113(43.7)	686 (45.3)
Age 45–64 y, *n* (%)	1376 (54.1)	797 (52.6)
Age ≥ 65 y, *n* (%)	56 (2.2)	32 (2.1)
Sex, *n* (%)		
Male	447 (17.6)	268 (17.7)
Female	2098 (82.4)	1247 (82.3)
Race, *n* (%)		
White	1974 (77.6)	1193 (78.7)
Black	453 (17.8)	261 (17.2)
Asian	30 (1.2)	15 (1.0)
Native Hawaiian or other Pacific Islander	10 (0.4)	5 (0.3)
American Indian or Alaska Native	37 (1.5)	22 (1.5)
Other	41 (1.6)	19 (1.3)
Mean (SD) BMI, kg/m^2^	36.2 (4.4)	36.2 (4.1)
BMI ≥ 30–<35, *n* (%)	960 (37.7)	547 (36.1)
BMI ≥ 35–<40, *n* (%)	876 (34.4)	596 (39.3)
BMI ≥ 40, *n* (%)	640 (25.1)	341 (22.5)
Hypertension, *n* (%)	646 (25.4)	367 (24.2)
Dyslipidemia, *n* (%)	1416 (55.6)	801 (52.9)
Type 2 diabetes, *n* (%)	333 (13.1)	169(11.2)
Complicated obesity, *n* (%)^a^	1596 (62.7)	991 (60.1)
History of anxiety, *n* (%)	103 (4.0)	63 (4.2)
History of depression, *n (%)*	305 (12.0)	193 (12.7)
Mean (SD) IDS-SR score^b^	6.9 (5.59)	6.6 (5.27)
No depression (IDS-SR score ≤ 13), *n* (%)	1907 (88.0)	1188 (89.0)
Mild depression (IDS-SR score 14–25), *n* (%)	244 (11.3)	138 (10.3)
Moderate depression (IDS-SR score 26–38), *n* (%)	16 (0.7)	8 (0.6)
Severe depression (IDS-SR score 39–48), *n* (%)	1 (0.05)	1 (0.07)
Tobacco use, *n* (%)	209 (8.2)	131 (8.6)
Alcohol use, *n* (%)	1080 (42.4)	661 (43.6)

All data are presented as *n* (%) unless otherwise noted

*BMI* body mass index, *IDS-SR* Inventory of Depressive Symptomatology, Subject-Rated, *NB* 32 mg naltrexone PR plus 360 mg bupropion PR (or 32 mg naltrexone immediate release plus 400 mg bupropion PR in study NB-201), *PR* prolonged release

a Complicated obesity was defined as BMI of ≥27 to ≤45 kg/m^2^ with controlled hypertension and/or dyslipidemia

b IDS-SR was not assessed in the phase 2 study (NB-201); NB, *n* = 2168; placebo, *n* = 1335. The 6 NB-treated (0.02%) and 2 placebo-treated (0.01%) subjects with baseline IDS-SR scores > 30 were eligible for participation in the phase 3 studies because their scores had been <30 at screening visits

**Table 3:** Summary of depression, anxiety, and sleep disorder-related adverse events based on grouped preferred terms in subjects with ≥1 event

Category^a^ Preferred term, *n* (%)	NB (*n* = 2545)	Placebo (*n* = 1515)	NB vs placebo *P*-value
Anxiety	138 (5.4)	50 (3.3)	0.029
Anxiety	108 (4.2)	43 (2.8)	0.118
Nervousness	13 (0.5)	2 (0.1)	0.071
Tension	10 (0.4)	2 (0.1)	0.374
Panic attack	6 (0.2)	3 (0.2)	0.912
Fear	2 (<0.1)	0	0.319
Panic reaction	1 (<0.1)	0	0.476
Generalized anxiety disorder	0	1 (<0.1)	0.087
Hyperventilation	0	0	–
Depression	47 (1.8)	41 (2.7)	0.014
Depression	24 (0.9)	23 (1.5)	0.088
Depressed mood	23 (0.9)	18 (1.2)	0.067
Dysthymic disorder	1 (0.1)	1 (<0.1)	0.613
Suicidal ideation	1 (<0.1)	3 (0.2)	0.045
Major depression	0	1 (<0.1)	0.087
Sleep disorders	322 (12.7)	119(7.9)	<0.001
Insomnia	233 (9.2)	89 (5.9)	<0.001
Sleep disorder	34 (1.3)	12 (0.8)	0.311
Abnormal dreams	25 (1.0)	6 (0.4)	0.114
Middle insomina	16 (0.6)	3 (0.2)	0.095
Poor quality sleep	8 (0.3)	2 (0.1)	0.336
Initial insomnia	6 (0.2)	4 (0.3)	0.225
Nightmare	7 (0.3)	1 (<0.1)	0.615
Sleep apnea syndrome	5 (0.2)	2 (0.1)	0.594
Terminal insomnia	2 (<0.1)	2 (0.1)	0.604

32 mg naltrexone PR plus 360 mg bupropion PR (or 32 mg naltrexone immediate release plus 400 mg bupropion PR in study NB-201), *PR* prolonged release

a Only categories that met statistical significance (*P* < 0.05) are reported here. Frequencies were analyzed using Cochran-Mantel-Haenszel general association test controlling for study. Additional potential anxiety symptoms (preferred terms: stress, restlessness, acute stress disorder, social avoidant behavior, hypervigilance, or social phobia) were reported in 19 (0.7%) and 16 (1.1%) NB and placebo subjects, respectively (*P* = 0.231).Additional potential depression symptoms (preferred terms: irritability, middle insomnia, libido decreased, stress, poor quality sleep, tearfulness, psychomotor hyperactivity, apathy, crying, depressive symptom, anhedonia, bereavement reaction, emotional distress, loss of libido, or negative thoughts) were reported in 125 (4.9%) and 55 (3.6%) NB and placebo subjects, respectively (*P* = 0.154). Additional symptoms of somnolence (preferred terms: somnolence, sedation, hypersomnia) were reported in 33 (1.3%) and 12 (0.8%) NB and placebo subjects, respectively (*P* = 0.087)

**Table 4:** IDS-SR assessed treatment-emergent depressive and anxiety symptoms^a^

Symptoms^b^, *n* (%)	NB (*n* = 2482)	Placebo (*n* = 1430)
≥1 postbaseline score of ≥2		
Item 5, sadness	76 (3.5)	45 (3.4)
Item 6, irritability	93 (4.3)	46 (3.4)
Item 7, anxiety/tension	139 (6.4)	64 (4.8)
Item 18, suicidality	12 (0.6)	5 (0.4)
≥1 postbaseline score of ≥25	96 (4.4)	55 (4.1)
≥1 postbaseline score of ≥30 for subjects with baseline score ≥ 25	2 (0.1)	2 (0.1)

*IDS-SR* Inventory of Depressive Symptomatology, Subject-Rated, *NB* prolonged-release naltrexone 32 mg plus prolonged-release bupropion 360 mg (or 400 mg bupropion PR plus 32 mg naltrexone immediate release in study NB-201)

a IDS-SR was not assessed in the phase 2 study (NB-201)

b In the Phase 3 studies, subjects who reported treatment-emergent IDS-SR scores ≥ 2 on item 5 [sadness], item 6 [irritability], item 7 [anxiety/tension] or suicidality items, or a total score ≥ 25 (or ≥30 for subjects with a score ≥ 25 at screening) were further evaluated and, if indicated, referred to a psychologist or psychiatrist, as values that met these thresholds may have indicated the presence of a treatment emergent depressive or anxiety disorder
